# Dichlorido[1-(1,10-phenanthrolin-2-yl)-2-pyridone]cadmium(II)

**DOI:** 10.1107/S1600536808019740

**Published:** 2008-07-05

**Authors:** Jin-Min Li

**Affiliations:** a Chemistry and Chemical Engineering College, Shanxi Datong University, Datong 037008, People’s Republic of China

## Abstract

In the title mononuclear complex, [CdCl_2_(C_17_H_11_N_3_O)], the Cd^II^ ion assumes a distorted trigonal–bipyramidal coordination geometry. The pyridone plane is twisted out of the 1,10-phenanthroline mean plane by 43.8 (3)°. In the crystal structure, short inter­molecular distances [3.627 (4)–3.671 (4) Å] between the centroids of the six- and five-membered Cd-containing rings suggest the existence of π–π inter­actions, which link the mol­ecules into stacks along the *a* axis.

## Related literature

For a related structure, see Liu *et al.* (2008 ▶).
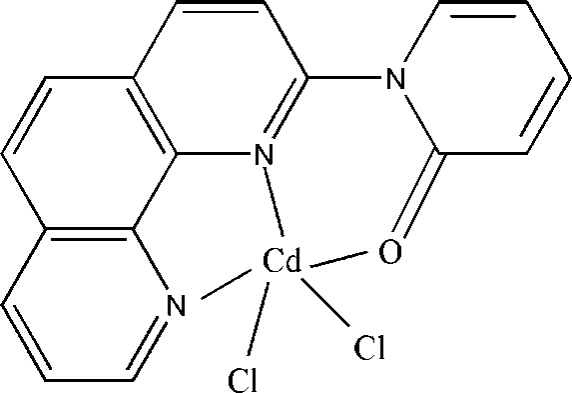


## Experimental

### 

#### Crystal data

[CdCl_2_(C_17_H_11_N_3_O)]
*M* *_r_* = 456.59
Monoclinic, 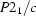

*a* = 7.5623 (13) Å
*b* = 14.105 (3) Å
*c* = 15.155 (3) Å
β = 97.728 (3)°
*V* = 1601.9 (5) Å^3^
*Z* = 4
Mo *K*α radiation
μ = 1.71 mm^−1^
*T* = 298 (2) K
0.11 × 0.06 × 0.05 mm

#### Data collection

Bruker SMART APEX CCD diffractometer
Absorption correction: multi-scan (*SADABS*; Sheldrick, 2008 ▶) *T*_min_ = 0.835, *T*_max_ = 0.920
9188 measured reflections
3476 independent reflections
2229 reflections with *I* > 2σ(*I*)
*R*_int_ = 0.071

#### Refinement

*R*[*F* ^2^ > 2σ(*F* ^2^)] = 0.071
*wR*(*F* ^2^) = 0.136
*S* = 1.05
3476 reflections
217 parameters
H-atom parameters constrained
Δρ_max_ = 1.15 e Å^−3^
Δρ_min_ = −0.83 e Å^−3^

### 

Data collection: *SMART* (Bruker, 1997 ▶); cell refinement: *SAINT* (Bruker, 1997 ▶); data reduction: *SAINT*; program(s) used to solve structure: *SHELXTL* (Sheldrick, 2008 ▶); program(s) used to refine structure: *SHELXTL*; molecular graphics: *SHELXTL*; software used to prepare material for publication: *SHELXTL* and local programs.

## Supplementary Material

Crystal structure: contains datablocks I, global. DOI: 10.1107/S1600536808019740/cv2426sup1.cif

Structure factors: contains datablocks I. DOI: 10.1107/S1600536808019740/cv2426Isup2.hkl

Additional supplementary materials:  crystallographic information; 3D view; checkCIF report

## Figures and Tables

**Table 1:** Centroid–centroid distances (Å) *Cg*1, *Cg*2 and *Cg*3 are the centroids of the rings Cd1/N2/N3/C8/C13, C8/C9/C11–C14 and N3/C13–C17, respectively.

*Cg*1⋯*Cg*2^i^	3.627 (4)
*Cg*2⋯*Cg*2^i^	3.631 (4)
*Cg*2⋯*Cg*3^ii^	3.671 (4)

Symmetry codes: (i) 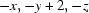; (ii) 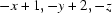.

**Fig. 1.**
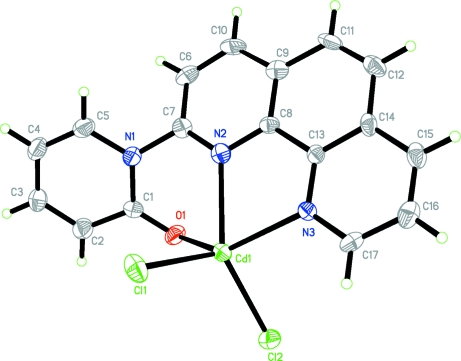
The molecular structure of the title complex showing the atom-numbering scheme and 30% probability displacement ellipsoids.

## supplementary crystallographic information

### Comment

Derivatives of 1,10-phenanthroline play an important role in modern coordination chemistry, and the complex with 1-(1,10-phenanthrolin-2-yl)-2-pyridone as bridging ligand and termial ligand has been reported (Liu et al.,  2008). Here I report the crystal structure of the title complex with 1-(1,10-phenanthrolin-2-yl)-2-pyridone as terminal ligand.

Fig. 1 shows the title coordination structure, revealing that the atom Cd is in a distorted trigonal bipyramidal environment. The dihedral angle between the pyridine ring plane and 1,10-phenanthroline ring system plane is 43.8 (3)°, which is smaller than that of the binuclear Cd^II^ complex (Liu et al., 2008). The crystal packing exhibits weak π–π stacking interactions involving symmetry-related neigbouring complexes, the relevant distances being Cg1···Cg2^i^ = 3.627 (4) Å and Cg1···Cg2^i^_perp_ = 3.407 Å and α = 4.70°; Cg2···Cg2^i^ = 3.631 (4) Å and Cg2···Cg2^i^_perp_ = 3.464 Å and α = 0.00°; Cg2···Cg3^ii^ =3.671 (4) Å and Cg2···Cg3^ii^_perp_ = 3.467 Å and α = 1.26° [symmetry codes: (i) -x, 2-y, -z; (ii) 1-x, 2-y, -z; Cg1, Cg2 and Cg3 are centroids of the Cd1/N2/N3/C8/C13, C8/C9/C11–C14 and N3/C13–C17 rings, respectively; Cgi···Cgj_perp_ is the perpendicular distance from ring Cgi to ring Cgj; α is the dihedral angle between ring plane Cgi and ring plane Cgj].

### Experimental

10 ml Methanol solution of 1-(1,10-phenanthrolin-2-yl)-2-pyridone (0.1620 g, 0.593 mmol) was added into 10 ml methanol solution containing CdCl_2_.2.5H_2_O (0.1352 g, 0.592 mmol) and the mixture was stirred for a few minutes. The colourless single crystals were obtained after the filtrate had been allowed to stand at room temperature for two weeks.

### Refinement

All H atoms were placed in calculated positions with C—H = 0.93 Å and refined as riding with *U*_iso_(H) = 1.2*U*_eq_(C).

### Crystal data

**Table:**

[CdCl_2_(C_17_H_11_N_3_O)]	*F*_000_ = 896
*M**_r_* = 456.59	*D*_x_ = 1.893 Mg m^−^^3^
Monoclinic, *P*2_1_/*c*	Mo *K*α radiation λ = 0.71073 Å
Hall symbol: -P 2ybc	Cell parameters from 970 reflections
*a* = 7.5623 (13) Å	θ = 2.7–18.8º
*b* = 14.105 (3) Å	µ = 1.71 mm^−^^1^
*c* = 15.155 (3) Å	*T* = 298 (2) K
β = 97.728 (3)º	Block, colourless
*V* = 1601.9 (5) Å^3^	0.11 × 0.06 × 0.05 mm
*Z* = 4	

### Data collection

**Table:**

Bruker SMART APEX CCD diffractometer	3476 independent reflections
Radiation source: fine-focus sealed tube	2229 reflections with *I* > 2σ(*I*)
Monochromator: graphite	*R*_int_ = 0.071
*T* = 298(2) K	θ_max_ = 27.0º
φ and ω scans	θ_min_ = 2.0º
Absorption correction: multi-scan(SADABS; Sheldrick, 2008)	*h* = −9→9
*T*_min_ = 0.835, *T*_max_ = 0.920	*k* = −17→17
9188 measured reflections	*l* = −12→19

### Refinement

**Table:**

Refinement on *F*^2^	Secondary atom site location: difference Fourier map
Least-squares matrix: full	Hydrogen site location: inferred from neighbouring sites
*R*[*F*^2^ > 2σ(*F*^2^)] = 0.071	H-atom parameters constrained
*wR*(*F*^2^) = 0.136	*w* = 1/[σ^2^(*F*_o_^2^) + (0.0492*P*)^2^] where *P* = (*F*_o_^2^ + 2*F*_c_^2^)/3
*S* = 1.05	(Δ/σ)_max_ = 0.001
3476 reflections	Δρ_max_ = 1.15 e Å^−^^3^
217 parameters	Δρ_min_ = −0.83 e Å^−^^3^
Primary atom site location: structure-invariant direct methods	Extinction correction: none

### Special details

**Table:**

Geometry. All e.s.d.'s (except the e.s.d. in the dihedral angle between two l.s. planes) are estimated using the full covariance matrix. The cell e.s.d.'s are taken into account individually in the estimation of e.s.d.'s in distances, angles and torsion angles; correlations between e.s.d.'s in cell parameters are only used when they are defined by crystal symmetry. An approximate (isotropic) treatment of cell e.s.d.'s is used for estimating e.s.d.'s involving l.s. planes.
Refinement. Refinement of *F*^2^ against ALL reflections. The weighted *R*-factor *wR* and goodness of fit *S* are based on *F*^2^, conventional *R*-factors *R* are based on *F*, with *F* set to zero for negative *F*^2^. The threshold expression of *F*^2^ > σ(*F*^2^) is used only for calculating *R*-factors(gt) *etc*. and is not relevant to the choice of reflections for refinement. *R*-factors based on *F*^2^ are statistically about twice as large as those based on *F*, and *R*- factors based on ALL data will be even larger.

### Fractional atomic coordinates and isotropic or equivalent isotropic displacement parameters (Å^2^)

**Table:**

	*x*	*y*	*z*	*U*_iso_*/*U*_eq_	
C1	0.1695 (9)	0.9518 (5)	0.3440 (5)	0.0366 (17)	
C2	0.1891 (10)	0.9363 (6)	0.4389 (5)	0.050 (2)	
H2	0.1560	0.8781	0.4603	0.060*	
C3	0.2543 (10)	1.0039 (6)	0.4973 (6)	0.052 (2)	
H3	0.2638	0.9922	0.5581	0.063*	
C4	0.3073 (11)	1.0914 (6)	0.4668 (6)	0.055 (2)	
H4	0.3551	1.1373	0.5072	0.066*	
C5	0.2892 (10)	1.1088 (5)	0.3801 (6)	0.049 (2)	
H5	0.3256	1.1672	0.3604	0.059*	
C6	0.1179 (10)	1.1614 (5)	0.2066 (6)	0.046 (2)	
H6	0.0853	1.2001	0.2514	0.056*	
C7	0.1882 (9)	1.0723 (5)	0.2263 (5)	0.0389 (18)	
C8	0.2128 (8)	1.0430 (5)	0.0795 (5)	0.0326 (16)	
C9	0.1472 (9)	1.1317 (5)	0.0533 (5)	0.0395 (18)	
C10	0.0974 (9)	1.1916 (5)	0.1209 (6)	0.047 (2)	
H10	0.0508	1.2515	0.1065	0.057*	
C11	0.1306 (10)	1.1576 (5)	−0.0390 (6)	0.047 (2)	
H11	0.0847	1.2168	−0.0566	0.057*	
C12	0.1796 (9)	1.0986 (6)	−0.1005 (6)	0.048 (2)	
H12	0.1671	1.1171	−0.1599	0.058*	
C13	0.2654 (8)	0.9785 (5)	0.0133 (4)	0.0305 (16)	
C14	0.2512 (9)	1.0072 (5)	−0.0751 (5)	0.0380 (18)	
C15	0.3059 (10)	0.9431 (6)	−0.1369 (5)	0.048 (2)	
H15	0.2987	0.9598	−0.1967	0.058*	
C16	0.3689 (10)	0.8574 (6)	−0.1090 (5)	0.049 (2)	
H16	0.4076	0.8150	−0.1494	0.059*	
C17	0.3761 (10)	0.8321 (5)	−0.0188 (5)	0.045 (2)	
H17	0.4158	0.7719	−0.0006	0.054*	
Cd1	0.31562 (7)	0.85238 (4)	0.18959 (4)	0.0400 (2)	
Cl1	0.5861 (3)	0.86363 (15)	0.29470 (14)	0.0613 (6)	
Cl2	0.2258 (3)	0.68924 (14)	0.15321 (14)	0.0580 (6)	
N1	0.2182 (7)	1.0428 (4)	0.3185 (4)	0.0379 (15)	
N2	0.2331 (7)	1.0138 (4)	0.1660 (4)	0.0369 (14)	
N3	0.3273 (7)	0.8923 (4)	0.0409 (4)	0.0330 (13)	
O1	0.1125 (7)	0.8907 (3)	0.2885 (3)	0.0460 (13)	

### Atomic displacement parameters (Å^2^)

**Table:**

	*U*^11^	*U*^22^	*U*^33^	*U*^12^	*U*^13^	*U*^23^
C1	0.034 (4)	0.034 (4)	0.042 (4)	0.001 (3)	0.007 (4)	0.005 (4)
C2	0.053 (5)	0.048 (5)	0.048 (5)	−0.009 (4)	0.004 (4)	−0.001 (4)
C3	0.065 (5)	0.056 (5)	0.036 (4)	0.001 (4)	0.007 (4)	−0.008 (4)
C4	0.069 (6)	0.050 (5)	0.045 (5)	−0.010 (4)	0.005 (5)	−0.014 (4)
C5	0.058 (5)	0.036 (4)	0.053 (5)	0.001 (4)	0.009 (5)	−0.006 (4)
C6	0.043 (4)	0.033 (4)	0.064 (6)	0.002 (3)	0.010 (4)	−0.013 (4)
C7	0.036 (4)	0.042 (4)	0.039 (4)	0.002 (3)	0.009 (4)	−0.001 (4)
C8	0.028 (4)	0.029 (4)	0.040 (4)	−0.002 (3)	0.004 (3)	0.001 (3)
C9	0.024 (4)	0.038 (4)	0.055 (5)	−0.003 (3)	0.000 (4)	0.003 (4)
C10	0.039 (4)	0.028 (4)	0.074 (6)	0.010 (3)	0.002 (4)	0.009 (4)
C11	0.050 (5)	0.035 (4)	0.054 (5)	−0.007 (4)	−0.008 (4)	0.022 (4)
C12	0.044 (5)	0.051 (5)	0.045 (5)	−0.011 (4)	−0.011 (4)	0.018 (4)
C13	0.027 (4)	0.033 (4)	0.030 (4)	−0.007 (3)	−0.001 (3)	0.000 (3)
C14	0.037 (4)	0.049 (5)	0.026 (4)	−0.010 (4)	−0.004 (3)	−0.002 (4)
C15	0.055 (5)	0.058 (5)	0.031 (4)	−0.024 (4)	0.007 (4)	0.001 (4)
C16	0.046 (5)	0.057 (5)	0.047 (5)	−0.012 (4)	0.013 (4)	−0.013 (5)
C17	0.052 (5)	0.029 (4)	0.054 (5)	−0.001 (3)	0.010 (4)	−0.010 (4)
Cd1	0.0511 (4)	0.0353 (3)	0.0328 (3)	0.0065 (3)	0.0029 (2)	0.0033 (3)
Cl1	0.0558 (12)	0.0765 (15)	0.0481 (12)	−0.0004 (11)	−0.0063 (10)	0.0119 (12)
Cl2	0.0922 (16)	0.0353 (10)	0.0463 (12)	−0.0013 (11)	0.0083 (12)	0.0006 (10)
N1	0.039 (4)	0.038 (3)	0.037 (4)	−0.003 (3)	0.005 (3)	−0.003 (3)
N2	0.028 (3)	0.041 (3)	0.041 (4)	0.001 (3)	0.001 (3)	0.000 (3)
N3	0.037 (3)	0.032 (3)	0.030 (3)	−0.001 (3)	0.003 (3)	0.002 (3)
O1	0.058 (3)	0.037 (3)	0.045 (3)	−0.010 (3)	0.012 (3)	−0.006 (3)

### Geometric parameters (Å, °)

**Table:**

C1—O1	1.239 (8)	C9—C11	1.436 (11)
C1—N1	1.404 (8)	C10—H10	0.9300
C1—C2	1.442 (10)	C11—C12	1.337 (11)
C2—C3	1.348 (10)	C11—H11	0.9300
C2—H2	0.9300	C12—C14	1.431 (10)
C3—C4	1.394 (11)	C12—H12	0.9300
C3—H3	0.9300	C13—N3	1.348 (8)
C4—C5	1.326 (11)	C13—C14	1.390 (9)
C4—H4	0.9300	C14—C15	1.403 (10)
C5—N1	1.375 (9)	C15—C16	1.347 (10)
C5—H5	0.9300	C15—H15	0.9300
C6—C10	1.356 (11)	C16—C17	1.407 (10)
C6—C7	1.382 (9)	C16—H16	0.9300
C6—H6	0.9300	C17—N3	1.329 (8)
C7—N2	1.310 (8)	C17—H17	0.9300
C7—N1	1.446 (9)	Cd1—N3	2.336 (6)
C8—N2	1.362 (8)	Cd1—O1	2.349 (5)
C8—C9	1.384 (9)	Cd1—N2	2.376 (6)
C8—C13	1.450 (9)	Cd1—Cl1	2.423 (2)
C9—C10	1.417 (11)	Cd1—Cl2	2.441 (2)
			
Cg1···Cg2^i^	3.627 (4)	Cg2···Cg3^ii^	3.671 (4)
Cg2···Cg2^i^	3.631 (4)		
			
O1—C1—N1	121.9 (7)	N3—C13—C14	122.7 (6)
O1—C1—C2	123.4 (7)	N3—C13—C8	117.9 (6)
N1—C1—C2	114.6 (7)	C14—C13—C8	119.4 (6)
C3—C2—C1	121.8 (7)	C13—C14—C15	117.7 (7)
C3—C2—H2	119.1	C13—C14—C12	119.9 (7)
C1—C2—H2	119.1	C15—C14—C12	122.4 (7)
C2—C3—C4	120.3 (8)	C16—C15—C14	119.5 (7)
C2—C3—H3	119.9	C16—C15—H15	120.2
C4—C3—H3	119.9	C14—C15—H15	120.3
C5—C4—C3	119.7 (8)	C15—C16—C17	119.9 (7)
C5—C4—H4	120.1	C15—C16—H16	120.1
C3—C4—H4	120.1	C17—C16—H16	120.1
C4—C5—N1	121.8 (8)	N3—C17—C16	121.4 (7)
C4—C5—H5	119.1	N3—C17—H17	119.3
N1—C5—H5	119.1	C16—C17—H17	119.3
C10—C6—C7	119.0 (7)	N3—Cd1—O1	132.29 (19)
C10—C6—H6	120.5	N3—Cd1—N2	70.5 (2)
C7—C6—H6	120.5	O1—Cd1—N2	72.21 (18)
N2—C7—C6	123.4 (7)	N3—Cd1—Cl1	118.71 (14)
N2—C7—N1	118.2 (6)	O1—Cd1—Cl1	97.58 (15)
C6—C7—N1	118.5 (7)	N2—Cd1—Cl1	102.58 (15)
N2—C8—C9	122.6 (6)	N3—Cd1—Cl2	93.15 (14)
N2—C8—C13	118.0 (6)	O1—Cd1—Cl2	100.09 (13)
C9—C8—C13	119.3 (7)	N2—Cd1—Cl2	144.10 (15)
C8—C9—C10	116.8 (7)	Cl1—Cd1—Cl2	113.26 (8)
C8—C9—C11	119.4 (7)	C5—N1—C1	121.7 (6)
C10—C9—C11	123.8 (7)	C5—N1—C7	117.2 (6)
C6—C10—C9	119.8 (7)	C1—N1—C7	121.0 (6)
C6—C10—H10	120.1	C7—N2—C8	118.4 (6)
C9—C10—H10	120.1	C7—N2—Cd1	125.8 (5)
C12—C11—C9	121.7 (7)	C8—N2—Cd1	115.4 (4)
C12—C11—H11	119.2	C17—N3—C13	118.8 (6)
C9—C11—H11	119.2	C17—N3—Cd1	123.5 (5)
C11—C12—C14	120.2 (7)	C13—N3—Cd1	117.4 (4)
C11—C12—H12	119.9	C1—O1—Cd1	113.3 (4)
C14—C12—H12	119.9		
			
O1—C1—C2—C3	178.9 (7)	N2—C7—N1—C1	−46.0 (9)
N1—C1—C2—C3	−1.8 (10)	C6—C7—N1—C1	135.6 (7)
C1—C2—C3—C4	−0.9 (12)	C6—C7—N2—C8	1.3 (10)
C2—C3—C4—C5	1.7 (13)	N1—C7—N2—C8	−177.0 (6)
C3—C4—C5—N1	0.4 (13)	C6—C7—N2—Cd1	−170.8 (5)
C10—C6—C7—N2	−1.5 (11)	N1—C7—N2—Cd1	10.9 (9)
C10—C6—C7—N1	176.8 (7)	C9—C8—N2—C7	0.1 (10)
N2—C8—C9—C10	−1.2 (10)	C13—C8—N2—C7	179.1 (6)
C13—C8—C9—C10	179.8 (6)	C9—C8—N2—Cd1	173.0 (5)
N2—C8—C9—C11	179.6 (6)	C13—C8—N2—Cd1	−8.0 (7)
C13—C8—C9—C11	0.7 (9)	N3—Cd1—N2—C7	−179.8 (6)
C7—C6—C10—C9	0.2 (11)	O1—Cd1—N2—C7	30.5 (5)
C8—C9—C10—C6	1.0 (10)	Cl1—Cd1—N2—C7	−63.5 (6)
C11—C9—C10—C6	−179.9 (7)	Cl2—Cd1—N2—C7	113.1 (5)
C8—C9—C11—C12	−1.0 (11)	N3—Cd1—N2—C8	7.9 (4)
C10—C9—C11—C12	179.9 (7)	O1—Cd1—N2—C8	−141.8 (5)
C9—C11—C12—C14	−0.2 (11)	Cl1—Cd1—N2—C8	124.2 (4)
N2—C8—C13—N3	1.6 (9)	Cl2—Cd1—N2—C8	−59.2 (5)
C9—C8—C13—N3	−179.4 (6)	C16—C17—N3—C13	1.8 (10)
N2—C8—C13—C14	−178.2 (6)	C16—C17—N3—Cd1	175.4 (5)
C9—C8—C13—C14	0.9 (9)	C14—C13—N3—C17	−0.3 (9)
N3—C13—C14—C15	−0.6 (10)	C8—C13—N3—C17	179.9 (6)
C8—C13—C14—C15	179.1 (6)	C14—C13—N3—Cd1	−174.4 (5)
N3—C13—C14—C12	178.2 (6)	C8—C13—N3—Cd1	5.9 (7)
C8—C13—C14—C12	−2.1 (10)	O1—Cd1—N3—C17	−140.4 (5)
C11—C12—C14—C13	1.8 (10)	N2—Cd1—N3—C17	179.1 (6)
C11—C12—C14—C15	−179.4 (7)	Cl1—Cd1—N3—C17	85.1 (5)
C13—C14—C15—C16	0.1 (10)	Cl2—Cd1—N3—C17	−33.7 (5)
C12—C14—C15—C16	−178.7 (7)	O1—Cd1—N3—C13	33.3 (5)
C14—C15—C16—C17	1.2 (11)	N2—Cd1—N3—C13	−7.2 (4)
C15—C16—C17—N3	−2.3 (11)	Cl1—Cd1—N3—C13	−101.2 (4)
C4—C5—N1—C1	−3.3 (11)	Cl2—Cd1—N3—C13	140.0 (4)
C4—C5—N1—C7	174.1 (7)	N1—C1—O1—Cd1	59.8 (7)
O1—C1—N1—C5	−176.9 (6)	C2—C1—O1—Cd1	−121.0 (6)
C2—C1—N1—C5	3.9 (9)	N3—Cd1—O1—C1	−104.1 (5)
O1—C1—N1—C7	5.9 (10)	N2—Cd1—O1—C1	−64.0 (5)
C2—C1—N1—C7	−173.4 (6)	Cl1—Cd1—O1—C1	36.8 (5)
N2—C7—N1—C5	136.6 (7)	Cl2—Cd1—O1—C1	152.2 (5)
C6—C7—N1—C5	−41.8 (9)		

Symmetry codes: (i) −*x*, −*y*+2, −*z*; (ii) −*x*+1, −*y*+2, −*z*.
